# Pathophysiological Concepts in Mild Traumatic Brain Injury: Diffusion Tensor Imaging Related to Acute Perfusion CT Imaging

**DOI:** 10.1371/journal.pone.0064461

**Published:** 2013-05-21

**Authors:** Zwany Metting, Leonardo Cerliani, Lars A. Rödiger, Joukje van der Naalt

**Affiliations:** 1 Department of Neurology, University Medical Center Groningen, Groningen, The Netherlands; 2 NeuroImaging Center, University of Groningen, Groningen, the Netherlands and Netherlands Institute for Neuroscience, Amsterdam, The Netherlands; 3 Department of Radiology, University Medical Center Groningen, Groningen, The Netherlands; University of Pittsburgh, United States of America

## Abstract

**Background:**

A subgroup of patients with mild traumatic brain injury (TBI) experiences residual symptoms interfering with their return to work. The pathophysiological substrate of the suboptimal outcome in these patients is a source of debate.

**Objective:**

To provide greater insight into the pathophysiological mechanisms of mild TBI.

**Methods:**

Diffusion tensor imaging (DTI) was performed during follow-up of 18 patients with mild TBI and compared with healthy control subjects. DTI data of the patient group were also compared with perfusion CT imaging in the acute phase of injury.

**Results:**

In patients with mild TBI, a trend was observed for a decreased fractional anisotropy (FA) in widespread bilateral frontal white matter areas with increased mean diffusivity (MD) in the parieto-temporal regions, compared to healthy control subjects. Cerebral blood volume (CBV) correlated significantly with FA in several white matter tracts including the corpus callosum, the internal capsule, the inferior fronto-occipital fascicle, the corticospinal tract, the superior and the inferior longitudinal fascicle.

**Conclusion:**

In mild TBI with normal conventional imaging significant associations between cerebral perfusion in the acute phase of injury and DTI analyses in the chronic phase of injury were discerned. The pathophysiological concept of these findings is being outlined.

## Introduction

A subgroup of patients with mild traumatic brain injury (TBI) continues to experience disabling symptoms that interfere with their return to work or resumption of social activities [1]. These symptoms not only cause a personal but also a socio-economical burden, since they often affect young patients in their twenties and thirties with full occupational status. In Europe TBI accounts for the highest number of total years lived with disability from trauma and belongs to the top three hospital costs per inhabitant [2]. It is of paramount importance to understand which mild TBI patients will develop cognitive disability in order to institute early rehabilitation. Various imaging techniques have demonstrated in patients with mild TBI both axonal injury and hemodynamic changes in mild TBI [3]. In a previous study we found frontal hypoperfusion with perfusion CT imaging in the acute phase after injury in patients with mild TBI and a normal conventional computed tomography (CT) [4]. Diffuse axonal injury (DAI), a major pathological substrate of TBI, can be visualized with diffusion tensor imaging (DTI), also in the mild TBI category [5]–[7]. The precise relation between hemodynamic changes in the acute phase and axonal injury remains speculative.

The purpose of this study was to examine the relation between measures of white matter integrity derived by DTI during follow-up and changes in perfusion derived by perfusion CT imaging in the acute phase after mild TBI, in order to explore possible common pathophysiological mechanisms.

## Materials and Methods

### Participants

Between 2005 and 2007 consecutive patients admitted with acute TBI were prospectively identified for enrolment in this study. Inclusion criteria were (1) age 18 to 65 years, (2) mild TBI defined as an initial GCS from 13 to 15 and (3) any period of posttraumatic amnesia (PTA). A cerebral non-contrast CT and a perfusion CT were performed on admission. In total 95 patients were included. Of these 76 patients had no abnormalities on conventional CT. This patient cohort was described earlier [4]. In the present study, only those patients were analyzed with a normal non-contrast CT on admission in whom also DTI was obtained during follow-up, because of posttraumatic complaints. Exclusion criteria comprised prior neurological or psychiatric disease, mental retardation, addiction to alcohol or drugs or inability for follow-up. Pregnancy, diabetes, nephropathy, presence of MRI-incompatible materials and contrast allergy were additional exclusion criteria. Written informed consent was obtained from patients, family or next of kin.

Two different healthy control groups were used, one (N = 25) obtained perfusion CT scans (10 men, 15 women; mean age 37 years (SD 12.2)); and another (N = 19) underwent magnetic resonance imaging (MRI) including DTI (11 men, 8 women; mean age 38.7 years (SD 10.4)). The healthy control subjects fulfilled the same exclusion criteria as the patient group and all gave written informed consent.

The study was approved by the Medical Ethical Committee of the University Medical Center Groningen.

### Imaging

#### Perfusion CT imaging

**Figure 1:**
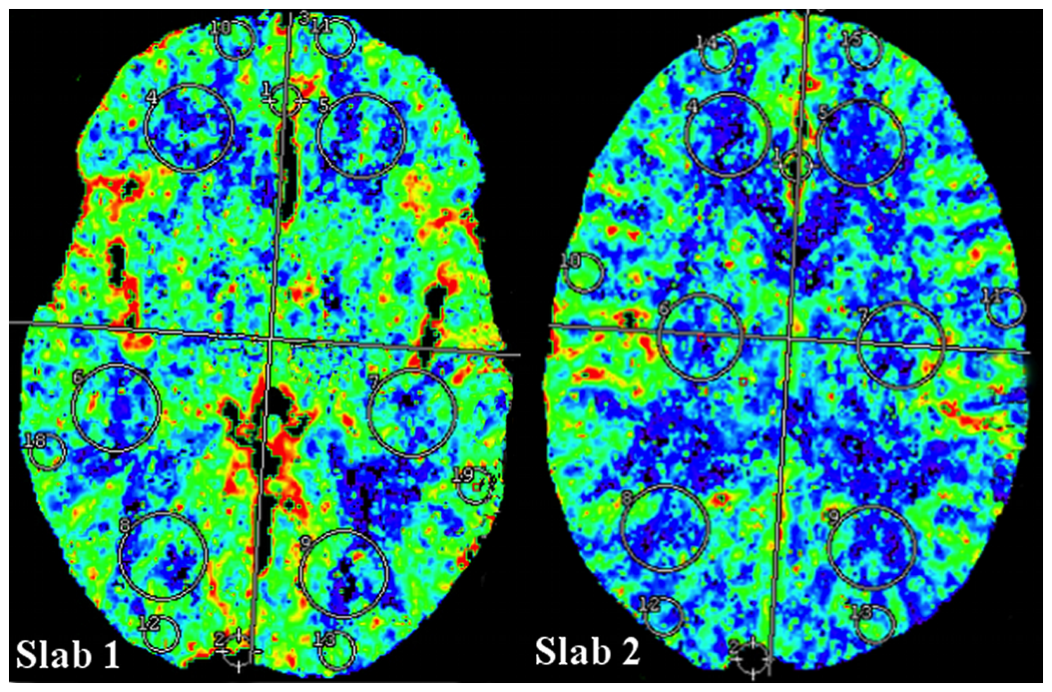
Two slabs with regions of interest (ROIs). In each slab six ROIs were placed in the white matter (WM) and six ROIs were placed in the cortical grey matter (GM). Slab 1 is lower slab, slab 2 is the upper slab.

CT scans were performed on a Siemens Somatom Sensation 64-row CT scanner (Siemens Medical Systems, Erlangen, Germany). The same scanning protocol was applied to all trauma patients and healthy control subjects. First a standard non-contrast CT of the brain was performed, followed by a perfusion CT. In our patient group, the non-contrast CT scans were all evaluated directly by a radiologist, in accordance with standard patient care. A central re-review of the non-contrast and perfusion CT scans were performed within a few days after trauma by an experienced neuroradiologist (L.R.). In this part of the study, we only analyzed data from patients without intracranial abnormalities on the non-contrast CT. Regarding perfusion CT imaging, two adjacent 14.4 mm thick slabs were positioned at the level of the third ventricle and at the level of the centrum semiovale. A 40-ml volume of a non-ionic iodinated contrast agent (Visipaque 270 mg/mL) was power-injected, followed by a saline chase. After 5 s delay, a dynamic scan was initiated with the following parameters: 80 kV, 100 mA, and 1 s per rotation for a duration of 46 s. Post-processing was performed by a neuroradiologist (L.R.) using TeraRecon perfusion software (TeraRecon Inc., San Mateo, CA, USA). A Gamma variate fitting curve was applied. The arterial input function was set in the anterior cerebral artery. The deconvolution algorithm produced two sets of colored parameter maps for cerebral blood flow (CBF), mean transit time (MTT) and cerebral blood volume (CBV). By using preset of regions of interest (ROIs) quantitative values for CBF, MTT and CBV were generated in the frontal, temporal and occipital white and grey matter (Figure 1).

#### Diffusion tensor imaging

Diffusion-weighted (DW) images were acquired using a PGSE EPI sequence (TR/TE = 4900/91 ms; NEX = 3) on a 1.5T Siemens Sonata (8-channel head coil) along 12 noncollinear directions (maximum gradient strength 40 mT/m; b-value 1000 sec/mm^2^; matrix 128×128; FOV 230×230 mm; no gap; reconstructed voxel size 0.9×0.9×5 mm^3^; 31 transverse slices). One image without diffusion gradients (b0) was also acquired. DW images were corrected for motion and eddy-currents using FSL software [8], [9] After b0 skull-stripping, FSL Fdt was used to perform diffusion tensor estimation and to calculate fractional anisotropy (FA) and mean diffusivity (MD) in each brain voxel. The resulting images were used to assess between-groups differences using Tract-Based Spatial Statistics (TBSS) [10].

### Statistical analysis

#### Perfusion CT

Statistical analyses were done using SPSS version 16 (SPSS Inc, Chicago, IL). Values for CBF, MTT and CBV from the corresponding ROIs in the lower and upper slab (see Figure 1) were averaged. Analysis of variance (ANOVA) was used to compare CBF, MTT and CBV values of patients with healthy control subjects. P-values were adjusted for age and gender.

#### The relation between diffusion tensor imaging (DTI) and perfusion CT

Between-groups differences in FA and MD values, as well as relationships between FA values and CBV values were estimated in two independent analyses using TBSS, included in FSL 4.1 (http://www.fmrib.ox.ac.uk/fsl). The steps of TBSS are detailed in the original paper by Smith and colleagues [10].

In summary:

1 Each subject's FA image is nonlinearly registered to the FMRIB58_FA template and checked for distortions generated by the alignment procedure.
2 The mean FA image is calculated, thresholded to an FA value of 0.2 and used for calculating the white matter skeleton, which estimates the location of the center of major fiber tracts common to all participants.
3 The white matter skeleton is overlaid onto each subject's registered FA image, and the maximum FA value along the normals to the skeleton is searched and dragged onto the skeleton.

TBSS has several advantages over whole-brain approaches used to estimate between-group differences in white matter integrity indices derived from diffusion tensor imaging: no smoothing is required either in the two-stages registration (steps 1. and 3.) or in the creation of the white matter skeleton, and the analyses are performed exclusively on the values which lie on the white matter skeleton. This increases the statistical power of the analysis and minimizes the chance that the results are driven by partial volume effects or confounding morphological differences (such as ventricles enlargement or atrophy).

4 Two separate analyses were perfor"list1" med. In the first, the mean FA and MD values for each voxel on the skeleton were compared between healthy control subjects and patients with mild TBI. In the second, performed only on the patient group, FA values for each voxel on the skeleton were regressed against cerebral perfusion parameters extracted from several ROIs in the brain (Figure 1). In both cases, parameter estimates were obtained from a general linear model (GLM) including also confound predictors for age and gender.
5 Inference was carried out by means of nonparametric permutation testing [11] implemented in FSL's randomise software. Five-thousands permutations were calculated for each analysis, in order to estimate the null distribution of either FA/MD differences, or of the correlation coefficients between FA and cerebral perfusion parameters. At the level of the single TBSS analysis, Threshold-Free Cluster Enhancement (TFCE) [12] was used to correct for multiple comparisons using the default values provided by the −T2 option of FSL randomize, which are optimized for TBSS analysis.

## Results

### Patient characteristics

In total twenty patients fulfilled the inclusion criteria for obtaining DTI. In two patients the technical data of the MRI scans were insufficient, hence 18 patients were enrolled in the study. Patient characteristics are displayed in Table 1. The mean time between injury and perfusion CT imaging was 3.6 hours (SD 1.3). The mean time between injury and DTI was 160 days (SD 109).

**Table 1:** Patient characteristics.

*Patient characteristics*(N = 18)	
Mean age in years, mean (SD)	38.0 (14.4)
Male	15 (84)
Traffic accidents	7 (39)
GCS, median	14
GCS categories 15	7 (39)
14	9 (50)
13	2 (11)
Duration PTA in hours, mean (SD)	5.75 (11.9)

Abbreviations: GCS = Glasgow Coma Score, PTA = posttraumatic amnesia.

Values are number (%) unless noted otherwise.

### Imaging

#### Perfusion CT

Perfusion CT values from the eighteen patients are displayed in Table 2 and compared with values from healthy control subjects. No significant differences between patients and healthy control subjects could be discerned. In all patients, perfusion CT scanning was completed without complications. No adverse reactions to the contrast material occurred.

**Table 2:** Cerebral perfusion in patients with mild traumatic brain injury compared to healthy controls.

		Areas	Patients(N = 18)	Controls(N = 25)
**CBF** **(ml • 100 g^−1^ • min^−1^)**	WM	**Global**	34.54±3.67	33.86±4.05
		Frontal	32.22±4.40	31.01±4.71
		Temporo-parietal	36.08±3.71	37.00±5.12
		Occipital	35.32±6.23	33.59±3.93
	GM	**Global**	42.90±5.04	45.47±4.17
		Frontal	43.13±5.43	46.71±5.51
		Temporo-parietal	46.14±6.04	48.00±5.62
		Occipital	39.41±5.99	41.74±5.08
**MTT** **(sec)**	WM	**Global**	4.05±0.68	4.47±0.64
		Frontal	3.93±0.74	4.42±0.72
		Temporo-parietal	4.03±0.54	4.28±0.58
		Occipital	4.18±0.83	4.70±0.70
	GM	**Global**	4.08±0.58	4.29±0.66
		Frontal	3.74±0.65	4.06±0.69
		Temporo-parietal	4.05±0.61	3.97±0.69
		Occipital	4.44±0.72	4.83±0.75
**CBV** **(ml • 100 g^−1^)**	WM	**Global**	2.30±0.24	2.25±0.26
		Frontal	2.08±0.29	2.00±0.26
		Temporo-parietal	2.40±0.28	2.42±0.29
		Occipital	2.41±0.28	2.33±0.31
	GM	**Global**	3.00±0.35	3.10±0.29
		Frontal	2.79±0.41	3.01±0.36
		Temporo-parietal	3.21±0.39	3.21±0.33
		Occipital	2.90±0.42	3.07±0.37

Cerebral blood flow (CBF), mean transit time (MTT) and cerebral blood volume (CBV) in different cerebral regions in the white matter (WM) and grey matter (GM).

#### Diffusion tensor imaging

**Figure 2:**
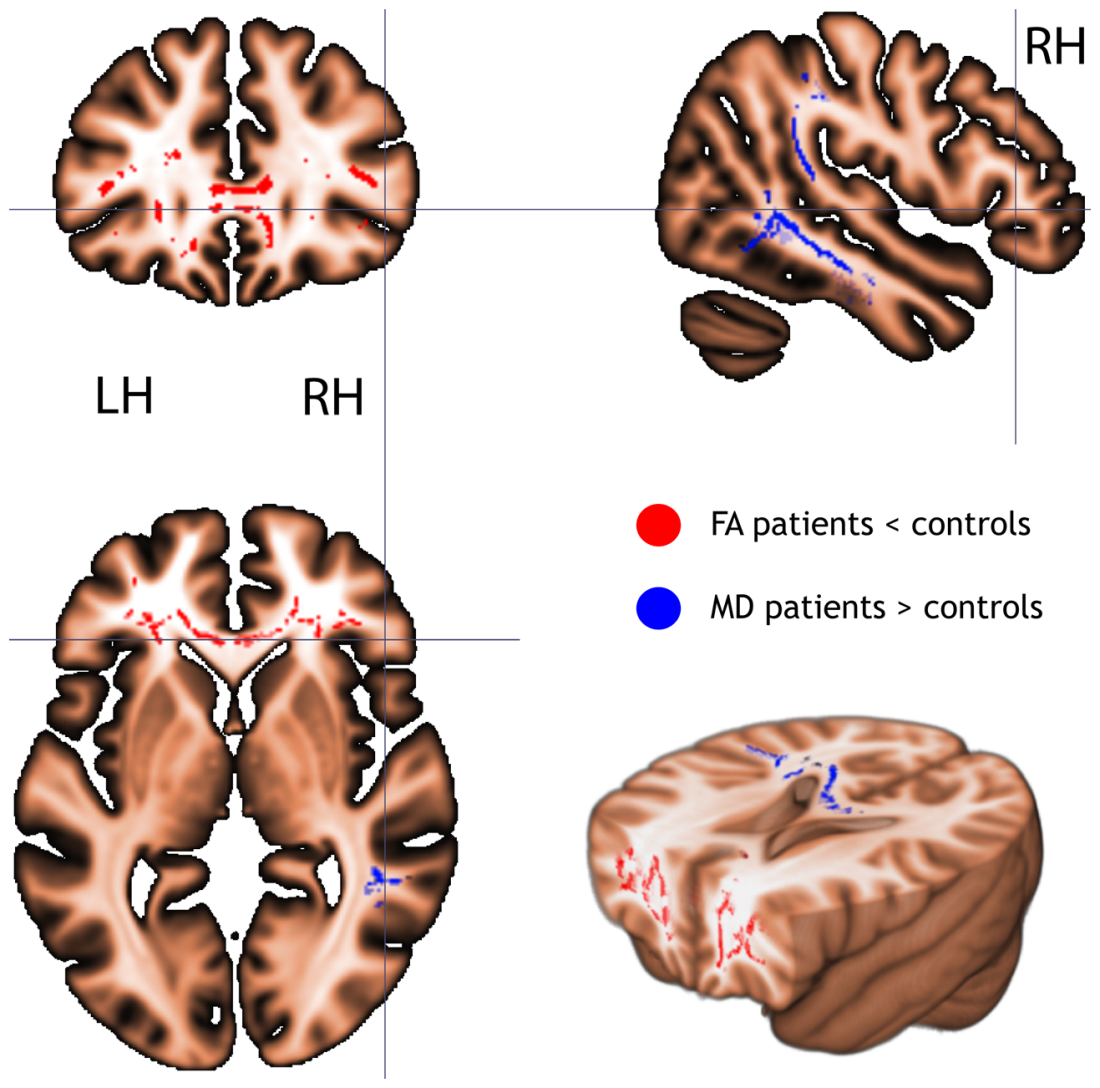
Fractional anisotropy (FA) and mean diffusivity (MD) in mild traumatic brain injury. FA and MD in mild traumatic brain injured patients compared to healthy control subjects. There was a trend for a lower FA (red; P<0.08 - TFCE corrected) and a higher MD (blue; P<0.07- TFCE corrected) in the patient group compared to the healthy control group.

In the patient group a trend was found for a decreased FA (P<0.08) in widespread bilateral white matter areas in the frontal regions, including the inferior fronto-occipital fasciculus and the white matter region underneath the right precuneus (Figure 2). The MD was increased, although not significantly, in the parieto-temporal regions (P<0.07) in the patient group compared to the healthy control subjects.

#### Relation of DTI with perfusion CT imaging

**Figure 3:**
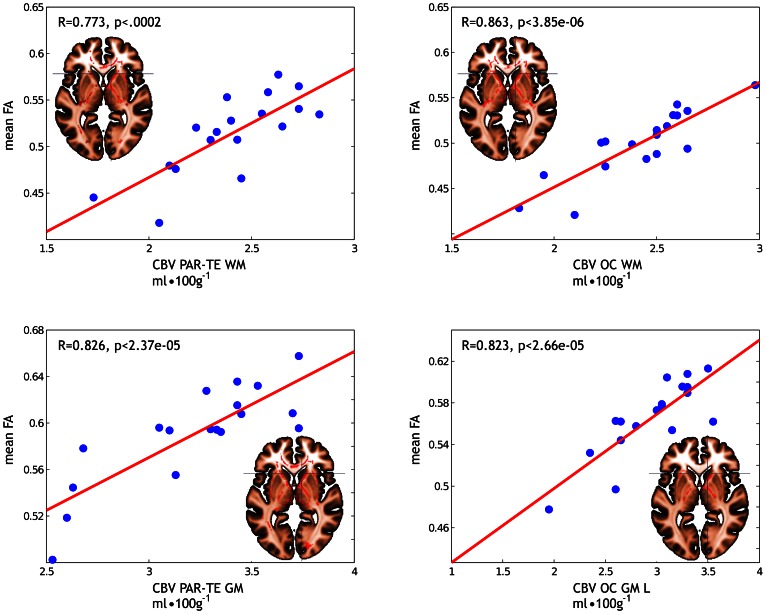
Fractional anisotropy (FA) in relation to cerebral blood volume (CBV) in mild traumatic brain injury. Significant (P<0.05 - TFCE corrected) correlations between FA and CBV in different cerebral areas in mild traumatic brain injured patients. The scatterplot reports the correlation between the CBV values and the mean FA calculated by averaging the FA values over all the voxels on the white matter skeleton where a significant (P<0.05 - TFCE corrected) effect was found in the voxelwise analysis. Abbreviations: PAR-TE = parieto-temporal, OC = occipital, WM = white matter, GM = grey matter, L = only on the left side.

A significant (P<0.05) positive correlation was present between CBV in white and grey matter areas in the parieto-temporal and occipital lobes and FA. Hence, a lower CBV was correlated with a lower FA (Figure 3 showing the voxel-by voxel correlations between CBV values and the mean FA). No negative correlations between CBV and FA could be discerned.

Significant correlations between CBV and FA were found in many different fiber tracts (Figure S1; providing all the axial slices at different Z coordinate levels), with a predominance for the corpus callosum including the anterior and posterior forceps, the corticospinal tract, the superior and inferior longitudinal fascicles, the external/extreme capsule and parts of the inferior fronto-occipital fascicle.

No significant correlations between FA and other perfusion parameters (MTT and CBF) were present.

## Discussion

This is the first study to examine the relationship between DTI and cerebral perfusion in patients with mild TBI. In patients with mild TBI and normal conventional imaging, a trend was observed towards DTI abnormalities in the chronic phase after injury. More importantly, these DTI findings were found to be associated with hemodynamic abnormalities assessed with perfusion CT imaging in the acute phase of injury. A challenging question is how to interpret the relation between these aforementioned findings.

In the acute phase after injury, hemodynamic changes are present in mild TBI [4]. Furthermore, several DTI studies identified subsequent white matter abnormalities in the chronic phase in patients with mild TBI [5]–[7], [13]–[16]. In general a decreased FA and an increased MD is seen after injury [5], [6], [13], [15], in accordance with our study. The most likely cause of these white matter changes is DAI. The precise chain of events over time in the occurrence of DAI remains poorly understood, especially in relation to the hemodynamic changes in acute phase after injury. Primary brain injury occurs at the moment of impact, with DAI as most important mechanism, even in mild TBI [17]. Secondary brain injury evolves in the hours after injury, mainly as a result of ischemia. Interestingly, Ueda and colleagues demonstrated in rat models that axonal damage is associated with vascular abnormalities in the early stage after injury. They suggested that injury forces might also damage the perivascular nerve networks, thereby contributing to the hemodynamic abnormalities [18]. Hence, the white matter abnormalities we observed in the present study might be secondary to comparable cerebral hemodynamic disturbances in the acute phase. Perfusion CT abnormalities in the acute phase of mild TBI were revealed in a previous study [4]. This study showed that CBV and CBF measurements had significant prognostic value suggesting a long-term consequence of these perfusion changes. The precise mechanisms responsible for hemodynamic alterations in TBI have not been completely elucidated. It is known that disruption of vascular tone following TBI is the result of effects exerted at the vascular smooth muscle and adventitial levels leading to perfusion changes that ultimately might result in tissue damage [19]. Furthermore, DAI results in impaired axonal transport and swelling, and this may further compromise small capillaries, thereby contributing to hemodynamic changes. The Wallerian degeneration of the axon and the consecutive structural reorganization may be reflected in changes in FA.

This is the first study to examine the relation between changes in cerebral perfusion in the acute phase of mild TBI and DTI abnormalities seen during follow-up. However, some limitations have to be acknowledged. First, the study group was small. Therefore the findings should be interpreted carefully and regarded as a preliminary observation. For the same reason, no subanalysis could be performed in relation to outcome. Second, this study concerns a subgroup of patients with mild TBI with complaints during follow-up. Nevertheless, we think that this is a relevant subanalysis regarding the unknown pathophysiological mechanisms in this patient category. Furthermore, the TBSS technique has advantages but also limitations over other approaches. In TBSS, the analyses are performed only in the white matter skeleton and differences in tract thickness cannot be detected. However, focussing only on the white matter regions lying at the center of the fiber tract has the advantage of greatly reducing the chance of partial volume effects. The white matter skeleton also does not include most of the outer white matter, where short-range U-shaped anatomical connections are located. However, results obtained in these regions are prone to be biased by misregistration across subjects, since the high intersubjective variability of the overlying sulcal pattern cannot be totally compensated by current registration algorithms. Finally, since TBSS analysis is performed voxelwise, the necessary correction for multiple comparisons may mask differences whose spatial extent is relatively limited. ROI-based analysis may be more sensitive than TBSS when strong hypotheses about the spatial location of the differences are available, and would benefit from a decrease in the noise due to averaging FA values across all ROI voxels. These factors may partly explain why in the present study the between-group differences in FA and MD only reached a trend to significance, although the size of the effect was comparable to that found in previous ROI-based studies [5], [20].

In summary, despite the small sample size that limits the overall generalizability of the results, with this study useful information is added to the discussion of the pathophysiological concept of mild TBI.

## Supporting Information

Figure S1 **Fractional anisotropy (FA) in relation to cerebral blood volume (CBV) in mild traumatic brain injury in all the axial slices of the whole white matter skeleton.** Significant (P<0.05 - TFCE corrected) correlations between FA and CBV in the parieto-temporal white matter (A), occipital white matter (B), parieto-temporal grey matter (C) and occipital grey matter on the left side (D) in mild traumatic brain injured patients. The scatterplot reports the correlation between the CBV values and the mean FA calculated FA values over all the voxels on the white matter skeleton where a significant (P<0.05 - TFCE corrected) effect was found in the voxelwise analysis. A. Correlations between CBV in the parieto-temporal white matter and FA were found in the corpus callosum as well as the anterior and posterior forceps, the cingulum bundle, the corticospinal tract, the anterior thalamic radiation, the superior longitudinal fascicle, the external/extreme capsule and parts of the inferior fronto-occipital fascicle and inferior longitudinal fascicle. B. Correlations between CBV in occipital white matter and FA were found in several locations of the corpus callosum, including the anterior forceps, in the dorsal section of the corticospinal tract, in some anterior parts of the external/extreme capsule. C. Correlations between CBV in the parieto-temporal grey matter and FA were found mostly in the corpus callosum, in the right superior longitudinal fascicle, in the dorsal portion of the corticospinal tract as well as in the right and, to a lesser extent, left internal capsule. D. Correlations between CBV in the occipital grey matter and FA were found predominantly in the left hemisphere, namely in some dorsal portion of the corticospinal tract, as well as in the internal and external/extreme capsule. Abbreviations: PAR-TE = parieto-temporal, OC = occipital, WM = white matter, GM = grey matter, L = only on the left side.
(TIFF)
