# Supplementary figures and images for: Pathophysiological Concepts in Mild Traumatic Brain Injury: Diffusion Tensor Imaging Related to Acute Perfusion CT Imaging
Source: PLoS One. 2013 May 21;8(5):e64461. doi: 10.1371/journal.pone.0064461 (PMC3660324; doi:10.1371/journal.pone.0064461)

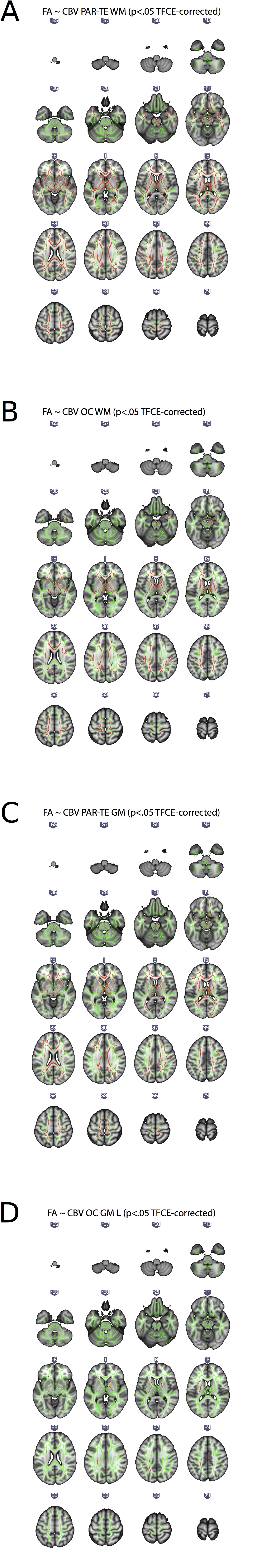

Supplement: Figure S1 — Fractional anisotropy (FA) in relation to cerebral blood volume (CBV) in mild traumatic brain injury in all the axial slices of the whole white matter skeleton. Significant (P<0.05 - TFCE corrected) correlations between FA and CBV in the parieto-temporal white matter (A), occipital white matter (B), parieto-temporal grey matter (C) and occipital grey matter on the left side (D) in mild traumatic brain injured patients. The scatterplot reports the correlation between the CBV values and the mean FA calculated FA values over all the voxels on the white matter skeleton where a significant (P<0.05 - TFCE corrected) effect was found in the voxelwise analysis. A. Correlations between CBV in the parieto-temporal white matter and FA were found in the corpus callosum as well as the anterior and posterior forceps, the cingulum bundle, the corticospinal tract, the anterior thalamic radiation, the superior longitudinal fascicle, the external/extreme capsule and parts of the inferior fronto-occipital fascicle and inferior longitudinal fascicle. B. Correlations between CBV in occipital white matter and FA were found in several locations of the corpus callosum, including the anterior forceps, in the dorsal section of the corticospinal tract, in some anterior parts of the external/extreme capsule. C. Correlations between CBV in the parieto-temporal grey matter and FA were found mostly in the corpus callosum, in the right superior longitudinal fascicle, in the dorsal portion of the corticospinal tract as well as in the right and, to a lesser extent, left internal capsule. D. Correlations between CBV in the occipital grey matter and FA were found predominantly in the left hemisphere, namely in some dorsal portion of the corticospinal tract, as well as in the internal and external/extreme capsule. Abbreviations: PAR-TE = parieto-temporal, OC = occipital, WM = white matter, GM = grey matter, L = only on the left side. (TIFF) [file pone.0064461.s001.tif]
